# A Data-Driven Scheme for Fault Detection of Discrete-Time Switched Systems

**DOI:** 10.3390/s21124138

**Published:** 2021-06-16

**Authors:** Hao Zhao, Hao Luo, Yunkai Wu

**Affiliations:** 1 Department of Control Science and Engineering, Harbin Institute of Technology, Harbin 150001, China; zhaohao316@hit.edu.cn; 2 School of Electronics and Information, Jiangsu University of Science and Technology, Zhenjiang 212003, China; wuyunkaischolar@just.edu.cn

**Keywords:** switched systems, fault detection, data-driven methods

## Abstract

This paper is concerned with the fault detection issue for a class of discrete-time switched systems via the data-driven approach. For the fault detection of switched systems, it is inevitable to consider the mode matching problem between the activated subsystem and the executed residual generator since the mode mismatching may cause a false fault alarm in all probability. Frequently, studies assume that the switching laws are available to the residual generator, by which the residual generator keeps the same mode as the system plant and then the mode mismatching is excluded. However, this assumption is conservative and impractical because many switching laws are hard to acquire in practical applications. This work focuses on the case of switched systems with unavailable switching laws. In view of the unavailability of switching information, the mode recognition is considered for the fault detection process and meanwhile, sufficient conditions are presented for the mode distinguishability. Moreover, a novel decision logic for the fault detection is proposed, based on which new algorithms are established for the data-driven realization. Finally, a benchmark case on a three-tank system is used to illustrate the feasibility and usefulness of the obtained results.

## 1. Introduction

With the increasing complication of industrial systems, high requirements are brought with the safety and reliability, which are critical to the stability and system performance. As is well known, fault detection has served as an effective tool to guarantee the safety and reliability of dynamic systems. Nowadays, the study on fault detection has drawn considerable attention from the literature [1,2,3,4,5,6,7,8,9]. The model-based fault detection takes up an important role in the fault detection field. By constructing the process model, the analysis and control issues have been excessively studied and numerous results of the model-based fault detection have been reported, see, e.g., [10,11,12,13,14] and the references therein. Recently, with the rapid development of communication technology, data-driven methodologies have been extensively addressed for the fault detection problems. Different from the model-based methodology, which requires a time-consuming and complicated modelling process from practical applications, the data-driven methodology gets rid of the modelling complexity and meanwhile sufficiently exploits the process data information. Therefore, great efforts have been made for the data-driven fault detection of various dynamical systems and practical applications, see, e.g., [15,16,17,18,19,20,21] and the references therein.

Moreover, increasing interest has been paid to the research on switched systems owing to their capacity in modelling the systems with switching behaviours [22,23,24,25,26,27,28,29,30,31]. As is known, a switched system usually consists of a finite number of distinct subsystems and a law governing the switching dynamics between these subsystems. The application of switched system models is rather widespread, such as flight control systems, communication systems, automotive industry and many other areas. Up to date, much research has been done on the analysis and synthesis of switched systems. For instance, the stability analysis and controller design are investigated for switched systems with constrained switching signals by exploiting the Lyapunov theory in [32,33,34,35]. Moreover, the switched systems with random switching signals are also considered and addressed to deal with the stability and stabilisation issues, such as [36,37,38].

As for the fault detection of switched systems, some results are available in the literature, see, e.g., [39,40,41,42]. It is worth mentioning that most of the existing results for the fault detection of switched systems are carried out on the assumption that the switching law is known and switching information is acquired to the residual generator. Based on this assumption, the modes between subsystem and residual generator are perfectly matched, which thus excludes the influence of system switching on the fault detection. In [43,44], the model-based approaches are investigated for switched systems with the determinate switching law, and in [45], a combination of switching observer and Bond Graph method is proposed while the switching signal is known. In [46,47], parity space method is applied to fault detection of the switching system, but the possibility that the switching signal is unknown is not considered. However, it is more frequent that the switching laws cannot be known or acquired in practice. In this case, the assumption of the switching information available to the residual generator is no longer applicable. Therefore, this work aims to cope with the fault detection for switched systems with unavailable switching laws.

Based on the discussion above, this paper focuses on achieving the data-driven fault detection for a class of discrete-time switched linear systems. Different from existing results, it is assumed that in this paper, the switching laws cannot be available to the mode-dependent residual generator. In this case, the mode mismatching between subsystem and residual generator may occur in the fault detection process, which may lead to incorrect residual signals and thus cause a false fault alarm. To handle this problem, the mode recognition is taken into account and sufficient criteria are proposed to ensure that the modes of the switched system are distinguishable. A novel decision logic including the mode recognition is developed in the data-driven fault detection, which avoids the false fault alarm caused by the mode mismatching and thus improves the accuracy of the fault detection. Then algorithms are presented to show the procedures of offline computation and online detection. The effectiveness and advantages of the developed method are demonstrated by the case study on a three-tank benchmark system. The paper is organised as follows. Section 2 provides the system descriptions and some preliminaries. In Section 3, the main results are presented including the sufficient criteria of mode distinguishability and novel algorithms for data-driven fault detection. Section 4 utilises the benchmark study of a three-tank model to illustrate the effectiveness and Section 5 concludes this work.

**Notations.** The notations used in this paper are standard. $R^{n}$ means the *n*-dimensional Euclidean space. ${\mathrm{RH}}_{\infty }^{m\times n}$ stands for the set of all m×n-dimensional real-rational transfer functions of stable systems. $N^{+}$ represents the set of all positive integers. $H_{2}$ defines the subspace of all signals ϖ(k) satisfying ϖ(k)=0 for t<0 and energy boundedness. ${∥\cdot ∥}_{2}$ is the Euclidean norm. $\sigma_{\max}\left(\cdot \right)$ defines the maximal singular value of a matrix. The superscript ‘‘T" represents the transpose of a matrix and the superscript ‘‘⊥" represents the orthogonal complement. The dimension of a matrix is assumed to be compatible with algebraic operations if it is not explicitly stated.

## 2. System Descriptions and Preliminaries

### 2.1. System Descriptions

This paper investigates the discrete-time switched linear system as below

(1) $$\begin{array}{c} \left\{\begin{array}{c} x\left(k+1\right)=A_{\sigma \left(k\right)}x\left(k\right)+B_{\sigma \left(k\right)}u\left(k\right)+\xi \left(k\right),\\ y\left(k\right)=C_{\sigma \left(k\right)}x\left(k\right)+D_{\sigma \left(k\right)}u\left(k\right)+\nu \left(k\right), \end{array}\right. \end{array}$$

where $x\left(k\right)\in R^{n_{x}},u\left(k\right)\in R^{n_{u}},y\left(k\right)\in R^{n_{y}}$ respectively denotes the system state, input and output signals. The symbol σ(k) defines the switching signal which is a piecewise constant function taking values in a finite set £={1,2,⋯,M}. $M(\in N^{+})$ stands for the number of subsystems. $\xi \left(k\right)$ and $\nu \left(k\right)$ stand for the system and measurement noise vectors which are normally distributed and statistically independent from x(0) and u(k). In the following, $A_{i},B_{i},C_{i},D_{i}$ are used to represent the system matrices $A_{\sigma \left(k\right)},B_{\sigma \left(k\right)},C_{\sigma \left(k\right)},D_{\sigma \left(k\right)}$ for $\sigma \left(k\right)=i\in £$.

For system (1) with $\xi \left(k\right)=0,\nu \left(k\right)=0$, its transfer matrix $G_{i}\left(z\right),i\in £$ describes the input-output behaviours of the system in the frequency domain as follows

$$\begin{array}{c} y\left(z\right)=G_{i}\left(z\right)u\left(z\right), \end{array}$$

where *z* is the complex variable. From the state space representation (1), there holds that

$$\begin{array}{c} G_{i}\left(z\right)=C_{i}{\left(zI-A_{i}\right)}^{-1}B_{i}+D_{i}, \end{array}$$

and usually, it is denoted that

$$\begin{array}{c} G_{i}\left(z\right)=\left[\begin{array}{cc} A_{i} & B_{i}\\ C_{i} & D_{i} \end{array}\right]. \end{array}$$

### 2.2. SKR-Based Residual Generators

Assume that $G_{i}\left(z\right),i\in £$ is a proper real-rational matrix. The left and right coprime factorisations are represented by

(2) $$\begin{array}{c} G_{i}\left(z\right)={\hat{M}}_{i}^{-1}\left(z\right){\hat{N}}_{i}\left(z\right)=N_{i}\left(z\right)M_{i}^{-1}\left(z\right), \end{array}$$

where $N_{i}\left(z\right)\in {\mathrm{RH}}_{\infty }^{n_{y}\times n_{u}},M_{i}\left(z\right)\in {\mathrm{RH}}_{\infty }^{n_{u}\times n_{u}},{\hat{N}}_{i}\left(z\right)\in {\mathrm{RH}}_{\infty }^{n_{y}\times n_{u}},{\hat{M}}_{i}\left(z\right)\in {\mathrm{RH}}_{\infty }^{n_{y}\times n_{y}}$. Then there exist $X_{i}\left(z\right)\in {\mathrm{RH}}_{\infty }^{n_{u}\times n_{u}},Y_{i}\left(z\right)\in {\mathrm{RH}}_{\infty }^{n_{u}\times n_{y}},{\hat{X}}_{i}\left(z\right)\in {\mathrm{RH}}_{\infty }^{n_{y}\times n_{y}},{\hat{Y}}_{i}\left(z\right)\in {\mathrm{RH}}_{\infty }^{n_{u}\times n_{y}}$ satisfying

$$\begin{array}{c} \left[\begin{array}{cc} X_{i}\left(z\right) & Y_{i}\left(z\right) \end{array}\right]\left[\begin{array}{c} M_{i}\left(z\right)\\ N_{i}\left(z\right) \end{array}\right]=I_{n_{u}\times n_{u}}, \end{array}$$

and

$$\begin{array}{c} \left[\begin{array}{cc} {\hat{M}}_{i}\left(z\right) & {\hat{N}}_{i}\left(z\right) \end{array}\right]\left[\begin{array}{c} {\hat{X}}_{i}\left(z\right)\\ {\hat{Y}}_{i}\left(z\right) \end{array}\right]=I_{n_{Y}\times n_{Y}}. \end{array}$$

The stable kernel representation (SKR) for each subsystem of system (1) is defined as follows.

**Definition** **1.** *For given system $G_{i}\left(z\right),i\in £$, if for any input u(z), it holds that*

$$\begin{array}{c} K_{i}\left[\begin{array}{c} u\left(z\right)\\ y\left(z\right) \end{array}\right]=0, \end{array}$$

*then the stable linear system $K_{i}$ is called a SKR of $G_{i}\left(z\right)$.*

It can be easily derived from the left coprime factorization in Equation (2) that

$$\begin{array}{c} K_{i}=\left[\begin{array}{cc} -{\hat{N}}_{i}\left(z\right) & {\hat{M}}_{i}\left(z\right) \end{array}\right]. \end{array}$$

Let $r_{i}\left(z\right)$ be the residual signal of $G_{i}\left(z\right),i\in £$. The SKR-based residual generator is given by

(3) $$\begin{array}{c} r_{i}\left(z\right)=K_{i}\left[\begin{array}{c} u\left(z\right)\\ y\left(z\right) \end{array}\right]=\left[\begin{array}{cc} -{\hat{N}}_{i}\left(z\right) & {\hat{M}}_{i}\left(z\right) \end{array}\right]\left[\begin{array}{c} u\left(z\right)\\ y\left(z\right) \end{array}\right]. \end{array}$$

### 2.3. K-Gap Metric

For the fault detection of systems with multiple modes, to measure the distance between the kernel subspaces of different modes is necessary. As is known, the K-gap metric works as an effective tool in measuring the distance between two kernel subspaces. Hence, this subsection introduces the concept of the K-gap metric. Firstly, the graph of subsystem $G_{i},i\in £$ is defined by

$$\begin{array}{c} K_{i}=\left\{\left[\begin{array}{c} u_{i}\\ y_{i} \end{array}\right]:\left[\begin{array}{cc} -{\hat{N}}_{i}\left(s\right) & {\hat{M}}_{i}\left(s\right) \end{array}\right]\left[\begin{array}{c} u_{i}\\ y_{i} \end{array}\right]=0,\left[\begin{array}{c} u_{i}\\ y_{i} \end{array}\right]\in H_{2}\right\}, \end{array}$$

which is a closed subsystem in $H_{2}$ representing the kernel subspace of $G_{i}$. The definition of the directed K-gap between two different graphs $K_{i}$ and $K_{j},i\neq j$ is described as below.

**Definition** **2** ([48])**.** *For any i≠j, the equation*

$$\begin{array}{c} {\vec{\delta }}_{k}\left(K_{i},K_{j}\right)=\underset{\left[\begin{array}{c} u_{i}\\ y_{i} \end{array}\right]\in K_{i}}{\sup}\underset{\left[\begin{array}{c} u_{j}\\ y_{j} \end{array}\right]\in K_{j}}{\inf}\frac{{∥\left[\begin{array}{c} u_{i}\\ y_{i} \end{array}\right]-\left[\begin{array}{c} u_{j}\\ y_{j} \end{array}\right]∥}_{2}}{{∥\left[\begin{array}{c} u_{i}\\ y_{i} \end{array}\right]∥}_{2}} \end{array}$$

*is called the directed K-gap of $K_{i}$ and $K_{j}$.*

Further, the K-gap metric of $K_{i}$ and $K_{j}$ is defined by

$$\begin{array}{c} \delta_{k}\left(K_{i},K_{j}\right)=\max\left\{{\vec{\delta }}_{k}\left(K_{i},K_{j}\right),{\vec{\delta }}_{k}\left(K_{j},K_{i}\right)\right\}. \end{array}$$

### 2.4. Structure of Data Matrices

In this subsection, the data structure is presented for the data-driven design of the fault detection in the latter text. For input u(k) and output y(k), define the following stacked data vectors

$$\begin{array}{c} u_{s}\left(k\right)=\left[\begin{array}{c} u\left(k\right)\\ \vdots \\ u\left(k+s-1\right) \end{array}\right],y_{s}\left(k\right)=\left[\begin{array}{c} y\left(k\right)\\ \vdots \\ y\left(k+s-1\right) \end{array}\right], \end{array}$$

where $s\in N^{+}$ is the data length. For a given $N\in N^{+}$, the Hankel matrices and the extended state vector are defined by

$$\begin{array}{cc} U_{k,s}= & \left[\begin{array}{cccc} u\left(k\right) & u\left(k+1\right) & \cdots & u\left(k+N-1\right)\\ u\left(k+1\right) & u\left(k+2\right) & \cdots & u\left(k+N\right)\\ \vdots & \vdots & \ddots & \vdots \\ u\left(k+s-1\right) & u\left(k+s\right) & \cdots & u\left(k+N+s-2\right) \end{array}\right]\\ = & \left[\begin{array}{ccc} u_{s}\left(k\right) & \cdots & u_{s}\left(k+N-1\right) \end{array}\right],\\ Y_{k,s}= & \left[\begin{array}{cccc} y\left(k\right) & y\left(k+1\right) & \cdots & y\left(k+N-1\right)\\ y\left(k+1\right) & y\left(k+2\right) & \cdots & y\left(k+N\right)\\ \vdots & \vdots & \ddots & \vdots \\ y\left(k+s-1\right) & y\left(k+s\right) & \cdots & y\left(k+N+s-2\right) \end{array}\right]\\ = & \left[\begin{array}{ccc} y_{s}\left(k\right) & \cdots & y_{s}\left(k+N-1\right) \end{array}\right],\\ X_{k,1}= & \left[\begin{array}{ccc} x\left(k\right) & \cdots & x\left(k+N-1\right) \end{array}\right]. \end{array}$$

Moreover, the past and future Hankel matrices are denoted by

$$\begin{array}{cc} Z_{p}= & \left[\begin{array}{c} U_{p}\\ Y_{p} \end{array}\right]=\left[\begin{array}{c} U_{k-s,s}\\ Y_{k-s,s} \end{array}\right],\\ Z_{f}= & \left[\begin{array}{c} U_{f}\\ Y_{f} \end{array}\right]=\left[\begin{array}{c} U_{k,s}\\ Y_{k,s} \end{array}\right]. \end{array}$$

The data-driven SKR for each subsystem of system (1) is presented based on the data structure.

**Definition** **3.** *For given system $G_{i}\left(z\right),i\in £$, if for any $u_{s}\left(k\right),x\left(0\right)$, it holds that*

$$\begin{array}{c} K_{i,d,s}\left[\begin{array}{c} u_{s}\left(k\right)\\ y_{s}\left(k\right) \end{array}\right]=0,\forall k\geq 0, \end{array}$$

*then the matrix $K_{i,d,s}$ is called a data-driven realisation of the SKR of $G_{i}\left(z\right)$.*

According to the formula (3), the data-driven residual generator is obtained as

(4) $$\begin{array}{c} r_{i}\left(k\right)=K_{i,d,s}\left[\begin{array}{c} u_{s}\left(k\right)\\ y_{s}\left(k\right) \end{array}\right]. \end{array}$$

## 3. Main Results

### 3.1. Problem Descriptions

**Figure 1:**
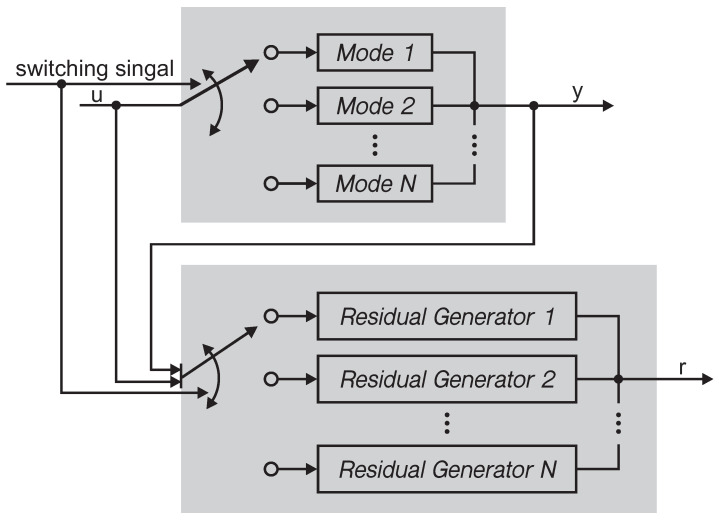
Schematic diagram of fault detection for switched systems with available switching laws.

This paper is concerned with the data-driven fault detection for discrete-time switched systems with unavailable switching laws. It is noteworthy that in the literature, most studies on the fault detection of switched systems are carried out on the assumption of the switching law availability. As shown in Figure 1, the available switching signal is transmitted to the system plant and residual generator simultaneously. In this case, the residual generator keeps the same mode with the system plant to generate the residual signal for fault detection, which in other words means that the system switching has no influence on the fault detection implementation.

**Figure 2:**
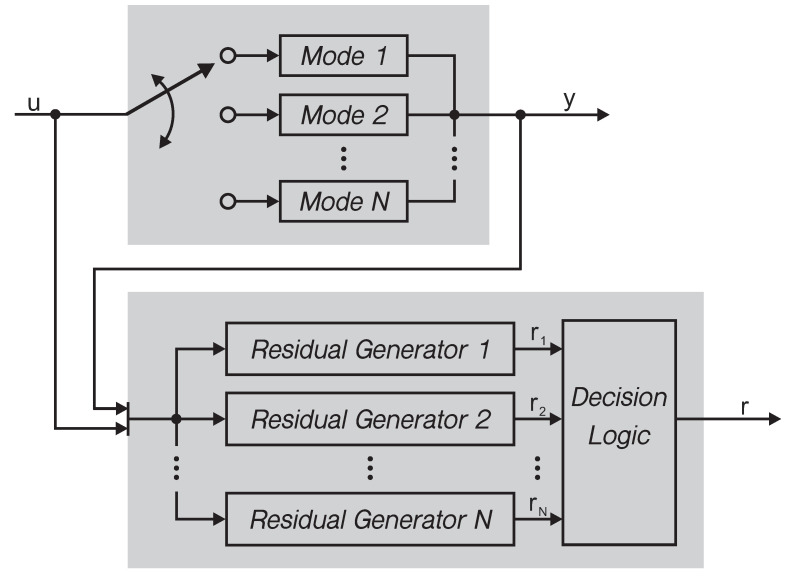
Schematic diagram of fault dection for switched systems proposed in this paper.

However, in engineering practice, it is more likely that the switching laws of a switched system cannot be known or acquired. For the switched system with unavailable switching laws, the fault detection becomes more complicated and challenging because of the possible mode mismatching between subsystem and residual generator. For example, when the system plant is activated in the mode *i*, the residual generator may work in the mode j(i≠j∈£) because of the unavailability of the switching laws. The mode mismatching between subsystem and residual generator may cause a terrible effect on the residual signal and thus the evaluation function. Even in the fault-free case, the evaluation function exceeds the threshold and a fault alarm is triggered. To handle this problem, this work establishes a set of decision logic as shown in Figure 2, in which the mode recognition is taken into account in the fault detection implementation. Thus, the mode mismatching influence is eliminated and the accuracy of the fault detection is improved. It is noteworthy that the mode recognition is carried out based on the distinguishability of different system modes, which will be discussed in detail in the following subsection.

### 3.2. Mode Distinguishability Conditions

To achieve the mode recognition, it is required that any two modes of the switched system are distinguishable. In the following, the discussion on the mode distinguishability is sufficiently presented. Consider the subsystem $G_{i},i\in £$ of switched system (1) with the associated SKR $K_{i}$. The definition of the cluster is recalled here for later use.

**Definition** **4** ([48])**.** *Given a scalar $r_{i}\in \left(0,1\right)$, the set*

$$\begin{array}{c} S_{i}\subseteq \left\{K:\delta_{k}\left(K,K_{i}\right)\leq r_{i}\right\} \end{array}$$

*is called $S_{i}$ cluster with the cluster centre $K_{i}$ and cluster radius $r_{i}$.*

On the basis of Definition 4, the definition of mode distinguishability is established as below.

**Definition** **5.** *We say that the modes $G_{i},i\in £$ of switched system (1) are distinguishable if for any $K\in S_{i}$, there does not exist $S_{j},j\neq i\in £$ such that $K\in S_{j}$.*

Here, we are in a position to propose a sufficient theorem for the mode distinguishability.

**Theorem** **1.** *Consider the SKR $K_{i}$ with the corresponding cluster $S_{i},i\in £$. $K_{i}$ is the cluster centre and $r_{i}$ is the cluster radius. If for any i≠j∈£, there holds that*

(5) $$\begin{array}{c} \delta_{k}\left(K_{i},K_{j}\right)>r_{i}+r_{j}, \end{array}$$

*then the modes of switched system (1) are distinguishable.*

**Proof** **of** **Theorem** **1.**  Firstly, suppose that the system modes are not distinguishable. According to Definition 5, it holds that for some $K\in S_{i}$, there exists mode j≠i∈£ such that $K\in S_{j}$. Due to $K\in S_{i}$ and $K\in S_{j}$, the following inequalities hold

(6) $$\begin{array}{c} \delta_{k}\left(K,K_{i}\right)\leq r_{i} \end{array}$$

and

(7) $$\begin{array}{c} \delta_{k}\left(K,K_{j}\right)\leq r_{j}. \end{array}$$

On the other hand, it is easy to get

$$\begin{array}{c} \delta_{k}\left(K,K_{j}\right)\geq \delta_{k}\left(K_{i},K_{j}\right)-\delta_{k}\left(K,K_{i}\right). \end{array}$$

Substituting condition (5) yields

$$\begin{array}{c} \delta_{k}\left(K,K_{j}\right)>r_{i}+r_{j}-r_{i}=r_{j}, \end{array}$$

which is contradictory to the inequality (7). Therefore, it can be concluded that the system modes are distinguishable, and the proof is completed. □

Theorem 1 presents a sufficient condition (5) to ensure that the modes of switched system are distinguishable. As shown in condition (5), the cluster radius $r_{i}$ is considered to be mode dependent, which gives rise to more freedom and thus less conservatism. By setting $r_{i}=r,\forall i\in £$, a degraded version of Theorem 1 could be derived as in the following corollary.

**Corollary** **1.** *Consider the SKR $K_{i}$ with the corresponding cluster $S_{i},i\in £$. $K_{i}$ is the cluster centre and r is the cluster radius. If for any i≠j∈£, there holds that*

$$\begin{array}{c} \delta_{k}\left(K_{i},K_{j}\right)>2r, \end{array}$$

*then the modes of switched system (1) are distinguishable.*

### 3.3. Mode Distinguishability Realisation

The above subsection constructs the sufficient conditions for the mode distinguishability of switched system (1). The essential issue is to ensure the K-gap metric of any two SKRs larger than the sum of their radii. Since this paper is concerned with the data-driven studies, this subsection aims to develop the data-driven realization of the K-gap metric. To this end, the normalised data-driven SKR is investigated in the first place.

**Definition** **6** ([49])**.** *If the data-driven SKR in Definition 3 satisfies $K_{i,d,s}K_{i,d,s}^{T}=I$, then it is called the normalised data-driven SKR.*

In the following, the normalised data-driven SKR is denoted as ${\bar{K}}_{i,d,s}$ to avoid confusion with the general version of SKR.

#### 3.3.1. Normalised Data-Driven SKR in the Open-Loop Case

For subsystem $G_{i},i\in £$, denote

$$\begin{array}{c} \Gamma_{i,s}=\left[\begin{array}{c} C_{i}\\ C_{i}A_{i}\\ \vdots \\ C_{i}A_{i}^{s-1} \end{array}\right],H_{i,u,s}=\left[\begin{array}{cccc} D_{i} & \cdots & 0 & 0\\ C_{i}B_{i} & \cdots & 0 & 0\\ \vdots & \ddots & \vdots & \vdots \\ C_{i}A_{i}^{s-2}B_{i} & \cdots & C_{i}B_{i} & D_{i} \end{array}\right]. \end{array}$$

The extended state space representation of subsystem $G_{i},i\in £$ is modelled by

(8) $$\begin{array}{c} Y_{k,s}^{i}=\Gamma_{i,s}X_{k,1}^{i}+H_{i,u,s}U_{k,s}^{i}+H_{i,\xi ,s}\Xi_{k,s}^{i}+V_{k,s}^{i}, \end{array}$$

where $H_{i,\xi ,s}$ has the same structure with $H_{i,u,s}$ and $\Xi_{k,s}^{i},V_{k,s}^{i}$ have the same structure with $U_{k,s}^{i}$ as defined in Section 2.4. $H_{i,\xi ,s}\Xi_{k,s}^{i}+V_{k,s}^{i}$ indicates the noise influence on the output. Rewrite (8) as

(9) $$\begin{array}{c} \left[\begin{array}{c} U_{k,s}^{i}\\ Y_{k,s}^{i} \end{array}\right]=\Psi_{i,s}\left[\begin{array}{c} U_{k,s}^{i}\\ X_{k,1}^{i} \end{array}\right]+\left[\begin{array}{c} 0\\ H_{i,\xi ,s}\Xi_{k,s}^{i}+V_{k,s}^{i} \end{array}\right],\Psi_{i,s}=\left[\begin{array}{cc} I & 0\\ H_{i,u,s} & \Gamma_{i,s} \end{array}\right]. \end{array}$$

Clearly, $\Psi_{i,s}^{⊥}$ is a data-driven realisation of the SKR $K_{i,d,s}$. The identification of $K_{i,d,s}=\Psi_{i,s}^{⊥}$ is built according to the following LQ decomposition

(10) $$\begin{array}{c} \left[\begin{array}{c} Z_{p}^{i}\\ U_{f}^{i}\\ Y_{f}^{i} \end{array}\right]=\left[\begin{array}{ccc} L_{i,11} & 0 & 0\\ L_{i,21} & L_{i,22} & 0\\ L_{i,31} & L_{i,32} & L_{i,33} \end{array}\right]\left[\begin{array}{c} Q_{i,1}\\ Q_{i,2}\\ Q_{i,3} \end{array}\right]. \end{array}$$

Since

$$\begin{array}{c} \left[\begin{array}{c} U_{f}^{i}\\ Y_{f}^{i} \end{array}\right]=\left[\begin{array}{cc} L_{i,21} & L_{i,22}\\ L_{i,31} & L_{i,32} \end{array}\right]\left[\begin{array}{c} Q_{i,1}\\ Q_{i,2} \end{array}\right]+\left[\begin{array}{c} 0\\ L_{i,33}Q_{i,3} \end{array}\right], \end{array}$$

and

$$\begin{array}{c} L_{i,33}Q_{i,3}=H_{i,\xi ,s}\Xi_{k,s}^{i}+V_{k,s}^{i}, \end{array}$$

it is implied that

$$\begin{array}{c} \Psi_{i,s}^{⊥}\left[\begin{array}{cc} L_{i,21} & L_{i,22}\\ L_{i,31} & L_{i,32} \end{array}\right]=\left[\begin{array}{cc} \Psi_{i,s,u}^{⊥} & \Psi_{i,s,y}^{⊥} \end{array}\right]\left[\begin{array}{cc} L_{i,21} & L_{i,22}\\ L_{i,31} & L_{i,32} \end{array}\right]=0. \end{array}$$

Performing the singular value decomposition of $K_{i,d,s}=\Psi_{i,s}^{⊥}=\left[\begin{array}{cc} \Psi_{i,s,u}^{⊥} & \Psi_{i,s,y}^{⊥} \end{array}\right]$ yields

$$\begin{array}{c} K_{i,d,s}=U_{i}\Sigma_{i}V_{i}^{T}=U_{i}\left[\begin{array}{cc} \Sigma_{1,i} & 0 \end{array}\right]\left[\begin{array}{c} V_{1,i}^{T}\\ V_{2,i}^{T} \end{array}\right]. \end{array}$$

As a consequence, the normalised data-driven SKR ${\bar{K}}_{i,d,s}$ in the open-loop case is obtained as

(11) $$\begin{array}{c} {\bar{K}}_{i,d,s}=V_{1,i}^{T}. \end{array}$$

#### 3.3.2. Normalised Data-Driven SKR in the Closed-Loop Case

For switched system (1), consider the feedback control system given by

(12) $$\begin{array}{c} \left\{\begin{array}{c} \tilde{x}\left(k+1\right)={\tilde{A}}_{i}\tilde{x}\left(k\right)+{\tilde{B}}_{i}\left(w\left(k\right)-y\left(k\right)\right),\\ u\left(k\right)={\tilde{C}}_{i}\tilde{x}\left(k\right)+{\tilde{D}}_{i}\left(w\left(k\right)-y\left(k\right)\right), \end{array}\right. \end{array}$$

where $\tilde{x}\left(k\right)$ is the controller’s state and $w\left(k\right)$ is the tracking reference. Suppose that the closed-loop of the *i*-th subsystem is well posed and internally stabilised by $K_{i}\left(z\right)=\left[{\tilde{A}}_{i},{\tilde{B}}_{i},{\tilde{C}}_{i},{\tilde{D}}_{i}\right]$. A similar representation as Formula (8) can be derived as below

(13) $$\begin{array}{c} U_{k,s}^{i}={\tilde{\Gamma }}_{i,s}{\tilde{X}}_{k,1}^{i}+{\tilde{H}}_{i,u,s}W_{k,s}^{i}-{\tilde{H}}_{i,u,s}Y_{k,s}^{i}, \end{array}$$

where ${\tilde{\Gamma }}_{i,s}$ and ${\tilde{H}}_{i,u,s}$ are composed of $K_{i}\left(z\right)=\left[{\tilde{A}}_{i},{\tilde{B}}_{i},{\tilde{C}}_{i},{\tilde{D}}_{i}\right]$ and have the similar structure with $\Gamma_{i,s}$ and $H_{i,u,s}$, respectively. For the *i*-th controller, ${\tilde{X}}_{k,1}^{i}=\left[\begin{array}{ccc} \tilde{x}\left(k\right) & \cdots & \tilde{x}\left(k+N-1\right) \end{array}\right]$ and $W_{k,s}^{i}$ is a Hankel matrix composed of vector $w\left(k\right)$. By substituting Equation (13) into Equation (8), the following formula holds

(14) $$\begin{array}{c} T_{i,s}Y_{k,s}^{i}=\Gamma_{i,s}X_{k,1}^{i}+H_{i,u,s}{\tilde{\Gamma }}_{i,s}{\tilde{X}}_{k,1}^{i}+H_{i,u,s}{\tilde{H}}_{i,u,s}W_{k,s}^{i}+H_{i,\xi ,s}\Xi_{k,s}^{i}+V_{k,s}^{i}, \end{array}$$

where $T_{i,s}=I+H_{i,u,s}{\tilde{H}}_{i,u,s}$. Note that the well-posedness of the *i*-th closed-loop guarantees the invertibility of matrix $T_{i,s}$. By denoting

$$\begin{array}{c} M_{k,s}^{i}=U_{k,s}^{i}+{\tilde{H}}_{i,u,s}Y_{k,s}^{i}={\tilde{\Gamma }}_{i,s}{\tilde{X}}_{k,1}^{i}+{\tilde{H}}_{i,u,s}W_{k,s}^{i}, \end{array}$$

the Formula (14) can be rewritten as

(15) $$\begin{array}{c} Y_{k,s}^{i}=T_{i,s}^{-1}\Gamma_{i,s}X_{k,1}^{i}+T_{i,s}^{-1}H_{i,u,s}M_{k,s}^{i}+T_{i,s}^{-1}\left(H_{i,\xi ,s}\Xi_{k,s}^{i}+V_{k,s}^{i}\right). \end{array}$$

Define

$$\begin{array}{cc} {\tilde{Z}}_{p}^{i}= & \left[\begin{array}{c} M_{p}^{i}\\ Y_{p}^{i} \end{array}\right],{\tilde{Z}}_{f}^{i}=\left[\begin{array}{c} M_{f}^{i}\\ Y_{f}^{i} \end{array}\right],\\ M_{p}^{i}= & U_{p}^{i}+{\tilde{H}}_{i,u,p}Y_{p}^{i},\\ M_{f}^{i}= & U_{f}^{i}+{\tilde{H}}_{i,u,f}Y_{f}^{i}. \end{array}$$

Similarly, by utilising the LQ decomposition

(16) $$\begin{array}{c} \left[\begin{array}{c} {\tilde{Z}}_{p}^{i}\\ M_{f}^{i}\\ Y_{f}^{i} \end{array}\right]=\left[\begin{array}{ccc} {\tilde{L}}_{i,11} & 0 & 0\\ {\tilde{L}}_{i,21} & {\tilde{L}}_{i,22} & 0\\ {\tilde{L}}_{i,31} & {\tilde{L}}_{i,32} & {\tilde{L}}_{i,33} \end{array}\right]\left[\begin{array}{c} {\tilde{Q}}_{i,1}\\ {\tilde{Q}}_{i,2}\\ {\tilde{Q}}_{i,3} \end{array}\right], \end{array}$$

and referring to [50], a data-driven SKR of $G_{i},i\in £$ can be derived as

$$\begin{array}{c} K_{i,d,s}=\left[\begin{array}{cc} {\tilde{K}}_{i,m,f} & {\tilde{K}}_{i,y,f}+{\tilde{K}}_{i,m,f}{\tilde{H}}_{i,u,f} \end{array}\right], \end{array}$$

where

$$\begin{array}{c} \left[\begin{array}{cc} {\tilde{K}}_{i,m,f} & {\tilde{K}}_{i,y,f} \end{array}\right]\left[\begin{array}{cc} {\tilde{L}}_{i,21} & {\tilde{L}}_{i,22}\\ {\tilde{L}}_{i,31} & {\tilde{L}}_{i,32} \end{array}\right]=0. \end{array}$$

Then consider the singular value decomposition

$$\begin{array}{c} K_{i,d,s}={\tilde{U}}_{i}{\tilde{\Sigma }}_{i}{\tilde{V}}_{i}^{T}={\tilde{U}}_{i}\left[\begin{array}{cc} {\tilde{\Sigma }}_{1,i} & 0 \end{array}\right]\left[\begin{array}{c} {\tilde{V}}_{1,i}^{T}\\ {\tilde{V}}_{2,i}^{T} \end{array}\right]. \end{array}$$

The normalised data-driven SKR ${\bar{K}}_{i,d,s}$ in the closed-loop case is given by

(17) $$\begin{array}{c} {\bar{K}}_{i,d,s}={\tilde{V}}_{1,i}^{T}. \end{array}$$

#### 3.3.3. Data-Driven Realisation of the K-Gap Metric

Based on the obtained normalised data-driven SKR ${\bar{K}}_{i,d,s}$ either in the open-loop or the closed-loop case, a theorem will be proposed to show how to realise the data-driven calculation of the K-gap metric between any two different modes. Before that, some preliminaries are restated here for the use of the deduction of the theorem.

**Definition** **7** ([51])**.** *Consider a truncation operator $\tau_{s}$ cutting off a time signal ϑ after s+1 sample times and is accordingly as*

$$\begin{array}{c} \tau_{s}:\vartheta \in L_{\left[0,\infty \right)}\to \vartheta_{tr}\in L_{\left[0,s\right)}. \end{array}$$

*A truncated or data-driven K-gap metric $\delta_{k_{d,s}}\left(K_{i},K_{j}\right)\left(i\neq j\in £\right)$ of two subsystems $G_{i},G_{j}$ with the corresponding SKRs $K_{i},K_{j}$ is defined by*

$$\begin{array}{c} \delta_{k_{d,s}}\left(K_{i},K_{j}\right)=\max\left\{{\vec{\delta }}_{k_{d,s}}\left(K_{i},K_{j}\right),{\vec{\delta }}_{k_{d,s}}\left(K_{j},K_{i}\right)\right\} \end{array}$$

*with*

$$\begin{array}{c} {\vec{\delta }}_{k_{d,s}}\left(K_{i},K_{j}\right)=\underset{z_{i,s}\in \tau_{s}K_{i}}{\sup}\underset{z_{j,s}\in \tau_{s}K_{j}}{\inf}\frac{{∥z_{i,s}-z_{j,s}∥}_{2}}{{∥z_{i,s}∥}_{2}}. \end{array}$$

The following lemma shows that the truncated result approximates to the real value of the K-gap metric as s→∞.

**Lemma** **1** ([51])**.** *For two subsystems $G_{i},G_{j}\left(i\neq j\in £\right)$ with the corresponding SKRs $K_{i},K_{j}$, the following formula is satisfied as s→∞,*

$$\begin{array}{c} \delta_{k_{d,s}}\left(K_{i},K_{j}\right)\to \delta_{k}\left(K_{i},K_{j}\right). \end{array}$$

**Theorem** **2.** *Consider two SKRs $K_{i},K_{j}\left(i\neq j\in £\right)$ with normalised data-driven SKRs ${\bar{K}}_{i,d,s},{\bar{K}}_{j,d,s}$. The data-driven realisation of the K-gap metric can be obtained by*

(18) $$\begin{array}{c} \delta_{k_{d,s}}\left(K_{i},K_{j}\right)=\max\left\{{\vec{\delta }}_{k_{d,s}}\left(K_{i},K_{j}\right),{\vec{\delta }}_{k_{d,s}}\left(K_{j},K_{i}\right)\right\}, \end{array}$$

*where*

(19) $$\begin{array}{c} {\vec{\delta }}_{k_{d,s}}\left(K_{i,d,s},K_{j,d,s}\right)=\sigma_{\max}\left({\bar{K}}_{i,d,s}^{T}-{\bar{K}}_{j,d,s}^{T}{\bar{K}}_{j,d,s}{\bar{K}}_{i,d,s}^{T}\right). \end{array}$$

**Proof** **of** **Theorem** **2.**  Due to the normalisation property of matrices ${\bar{K}}_{i,d,s},{\bar{K}}_{j,d,s}$, they respectively construct the orthonormal basis of the kernel spaces $K_{i},K_{j}$. The truncated kernel spaces are given by

$$\begin{array}{c} \tau_{s}K_{i}=C\left({\bar{K}}_{i,d,s}^{T}\right),\tau_{s}K_{j}=C\left({\bar{K}}_{j,d,s}^{T}\right). \end{array}$$

According to Definition 7,

(20) $$\begin{array}{cc} {\vec{\delta }}_{k_{d,s}}\left(K_{i},K_{j}\right)= & \underset{z_{i,s}\in C\left({\bar{K}}_{i,d,s}^{T}\right)}{\sup}\underset{z_{j,s}\in C\left({\bar{K}}_{j,d,s}^{T}\right)}{\inf}\frac{{∥z_{i,s}-z_{j,s}∥}_{2}}{{∥z_{i,s}∥}_{2}}\\ = & \underset{\alpha }{\sup}\underset{\beta }{\inf}\frac{{∥{\bar{K}}_{i,d,s}^{T}\alpha -{\bar{K}}_{j,d,s}^{T}\beta ∥}_{2}}{{∥{\bar{K}}_{i,d,s}^{T}\alpha ∥}_{2}}. \end{array}$$

It is obvious that with respect to β, the Equation (20) has only one minimal value in the global domain. Hence, let

(21) $$\begin{array}{c} \frac{\partial }{\partial \beta }\left\{{\left({\bar{K}}_{i,d,s}^{T}\alpha -{\bar{K}}_{j,d,s}^{T}\beta \right)}^{T}\left({\bar{K}}_{i,d,s}^{T}\alpha -{\bar{K}}_{j,d,s}^{T}\beta \right)\right\}=0. \end{array}$$

The solution of Equation (21) is calculated as

(22) $$\begin{array}{c} \beta ={\bar{K}}_{j,d,s}{\bar{K}}_{i,d,s}^{T}\alpha . \end{array}$$

Substituting (22) into Equation (20) gives rise to

(23) $$\begin{array}{c} {\vec{\delta }}_{k_{d,s}}\left(K_{i},K_{j}\right)=\underset{\alpha }{\sup}\frac{{∥\left({\bar{K}}_{i,d,s}^{T}-{\bar{K}}_{j,d,s}^{T}{\bar{K}}_{j,d,s}{\bar{K}}_{i,d,s}^{T}\right)\alpha ∥}_{2}}{{∥{\bar{K}}_{i,d,s}^{T}\alpha ∥}_{2}}, \end{array}$$

which thus implies the expression (19). □

### 3.4. Data-Driven Fault Detection

In this subsection, the data-driven realisation of the fault detection is presented for the switched system (1). For each subsystem $G_{i},i\in £$, the residual evaluation function is constructed by

(24) $$\begin{array}{c} J_{i}\left(k\right)={∥r_{i}\left(k\right)∥}_{2}^{2}=\underset{n=0}{\sum^{s-1}}r_{i}^{T}\left(k+n\right)r_{i}\left(k+n\right), \end{array}$$

and the threshold is set as

(25) $$\begin{array}{c} J_{i,th}=\underset{k}{\sup}J_{i}\left(k\right). \end{array}$$

The following Algorithm 1 is proposed to show how to determine the data-driven residual generator and the threshold for each subsystem based on the offline process data.
**Algorithm 1**Offline Data-Driven ProcedureStep 1:Collect the process data $u_{i},y_{i}$ of each subsystem $G_{i},i\in £$Step 2:Choose s,N and build the Hankel matrices $U_{k,s}^{i},Y_{k,s}^{i},Z_{p}^{i}$ for open-loop case
or ${\tilde{Z}}_{p}^{i},M_{f}^{i},Y_{f}^{i}$ for closed-loop caseStep 3:Perform LQ decomposition (10) or (16) and calculate the data-driven SKR $K_{i,d,s}$Step 4:Utilise the singular value decomposition to get the normalised data-driven SKR ${\bar{K}}_{i,d,s}$
in (11) or (17)Step 5:Calculate the K-gap metric $\delta_{k_{d,s}}\left(K_{i},K_{j}\right)$ of any two modes according to (18) and compare it
with given scalar λ>0
$\left\{\begin{array}{c} \mathrm{If}\;\delta_{k_{d,s}}\left(K_{i},K_{j}\right)>\lambda ,\forall i\neq j\in £,\;\mathrm{go}\;\mathrm{to}\;\mathrm{Step}\;6\\ \mathrm{If}\;\mathrm{there}\;\mathrm{exist}\;\mathrm{some}\;i\neq j\in £\;\mathrm{such}\;\mathrm{that}\;\delta_{k_{d,s}}\left(K_{i},K_{j}\right)\leq \lambda ,\;\mathrm{return}\;\mathrm{to}\;\mathrm{Step}\;1\;\mathrm{and}\;\mathrm{update}\;\mathrm{the}\;\mathrm{data} \end{array}\right.$Step 6:Construct the data-driven residual generator according to (4)Step 7:Run the evaluation function (24) and set the threshold $J_{i,th}$

By virtue of the constructed residual generator in Algorithm 1, the online residual signal $r_{i}\left(k\right)$ and evaluation function $J_{i}\left(k\right)$ can be obtained with the online data. By comparing $J_{i}\left(k\right)$ with $J_{i,th}$ for each mode, the decision logic is implemented as follows.

(26) $$\begin{array}{c} \left\{\begin{array}{c} \mathrm{If}\;J_{i}\left(k\right)>J_{i,th},\forall i\in £\Rightarrow \;\mathrm{faulty}\\ \mathrm{If}\;\mathrm{there}\;\mathrm{exists}\;i\in £\;\mathrm{such}\;\mathrm{that}\;J_{i}\left(k\right)\leq J_{i,th}\Rightarrow \;\mathrm{fault}-\mathrm{free} \end{array}\right. \end{array}$$

Then the online procedure of the data-driven fault detection for switched system (1) is described in the following Algorithm 2.
**Algorithm 2**Online Fault DetectionStep 1:Collect the online process data $u_{s}\left(k\right),y_{s}\left(k\right)$Step 2:Run the residual generator (4) with each SKR $K_{i,d,s},i\in £$Step 3:Obtain the residual signal $r_{i}\left(k\right)$ and the evaluation function $J_{i}\left(k\right)$ according to (24)Step 4:Implement the decision logic (26)

## 4. Benchmark Study

**Figure 3:**
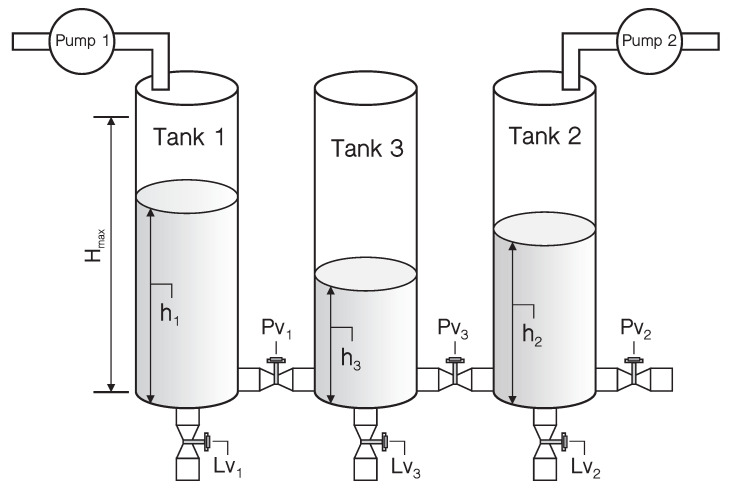
Structure of the three-tank system.

The benchmark study on a three-tank system has been demonstrated in this section, which can be regarded as the prototype for many industrial systems. As sketched in Figure 3, a basic structure of three-tank system includes three water tanks, two connecting pipes, four drain pipes and two water pumps. All the six pipes can be opened or closed by the adjustable ball valves PV${}_{1}$, PV${}_{2}$, PV${}_{3}$, LV${}_{1}$, LV${}_{2}$, LV${}_{3}$ through a controller or manual adjustment. Through Pump 1 and Pump 2, water is pumped into Tank 1 and Tank 2 with the incoming mass flow rates Q${}_{1}$ and Q${}_{2}$, respectively. The liquid levels h${}_{1}$, h${}_{2}$ and h${}_{3}$ of three-tank can be measured by the liquid level sensors with the maximum allowable water level h${}_{max}$. The water pump will stop working when the liquid level is higher then h${}_{max}$. The incoming mass flow rates of two pumps Q${}_{1}$ and Q${}_{2}$ and the liquid levels h${}_{1}$, h${}_{2}$ and h${}_{3}$ are chosen as system inputs and measured outputs, respectively.

In this work, the three-tank system operates around the working point h${}_{1}$ = 45 cm, h${}_{2}$ = 15 cm and h${}_{3}$ = 30 cm. Different combinations of the adjustable ball valve’s state compose the different modes of the system. Consider the three-tank system with three modes as shown in Table 1. Specifically, the mathematical model for each mode is described as follows.

For the first mode, open the valves PV${}_{1}$, PV${}_{2}$, PV${}_{3}$ fully, open the valve LV${}_{1}$ by 80% and close the valves LV${}_{2}$,LV${}_{3}$. The dynamics of this system mode is formulated by

$$\begin{array}{cc} A{\dot{h}}_{1}= & Q_{1}-\alpha_{1}s_{n}sgn\left(h_{1}-h_{3}\right)\sqrt{2g|h_{1}-h_{3}|}-80\%\beta_{1}s_{l}\sqrt{2gh_{1}},\\ A{\dot{h}}_{2}= & Q_{2}+\alpha_{3}s_{n}sgn\left(h_{3}-h_{2}\right)\sqrt{2g|h_{3}-h_{2}|}-\alpha_{2}s_{n}\sqrt{2gh_{2}},\\ A{\dot{h}}_{3}= & \alpha_{1}s_{n}sgn\left(h_{1}-h_{3}\right)\sqrt{2g|h_{1}-h_{3}|}-\alpha_{3}s_{n}sgn\left(h_{3}-h_{2}\right)\sqrt{2g|h_{3}-h_{2}|}. \end{array}$$

For the second mode, open the valves PV${}_{1}$, PV${}_{2}$, PV${}_{3}$ fully and close the valves LV${}_{1}$, LV${}_{2}$, LV${}_{3}$. The dynamics of this system mode is given by

$$\begin{array}{cc} A{\dot{h}}_{1}= & Q_{1}-\alpha_{1}s_{n}sgn\left(h_{1}-h_{3}\right)\sqrt{2g|h_{1}-h_{3}|},\\ A{\dot{h}}_{2}= & Q_{2}+\alpha_{3}s_{n}sgn\left(h_{3}-h_{2}\right)\sqrt{2g|h_{3}-h_{2}|}-\alpha_{2}s_{n}\sqrt{2gh_{2}},\\ A{\dot{h}}_{3}= & \alpha_{1}s_{n}sgn\left(h_{1}-h_{3}\right)\sqrt{2g|h_{1}-h_{3}|}-\alpha_{3}s_{n}sgn\left(h_{3}-h_{2}\right)\sqrt{2g|h_{3}-h_{2}|}. \end{array}$$

For the third mode, open the valves PV${}_{1}$, PV${}_{3}$ fully, open the valves LV${}_{1}$, LV${}_{2}$ by 20%, 80%, respectively and close the valves PV${}_{2}$, LV${}_{3}$. Correspondingly, the dynamics of this mode is described by

$$\begin{array}{cc} A{\dot{h}}_{1}= & Q_{1}-\alpha_{1}s_{n}sgn\left(h_{1}-h_{3}\right)\sqrt{2g|h_{1}-h_{3}|}-20\%\beta_{1}s_{l}\sqrt{2gh_{1}},\\ A{\dot{h}}_{2}= & Q_{2}+\alpha_{3}s_{n}sgn\left(h_{3}-h_{2}\right)\sqrt{2g|h_{3}-h_{2}|}-80\%\beta_{2}s_{l}\sqrt{2gh_{2}},\\ A{\dot{h}}_{3}= & \alpha_{1}s_{n}sgn\left(h_{1}-h_{3}\right)\sqrt{2g|h_{1}-h_{3}|}-\alpha_{3}s_{n}sgn\left(h_{3}-h_{2}\right)\sqrt{2g|h_{3}-h_{2}|}. \end{array}$$

**Figure 4:**
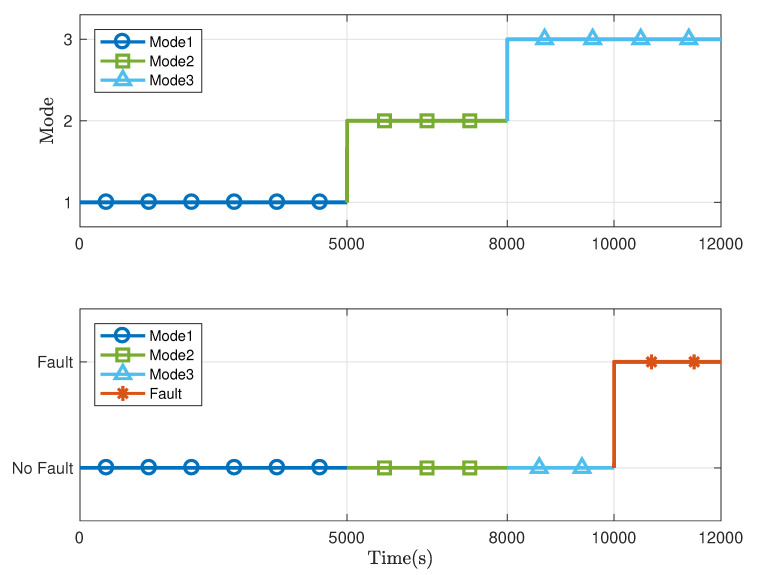
Switching law and fault signal of the three-tank system.

**Figure 5:**
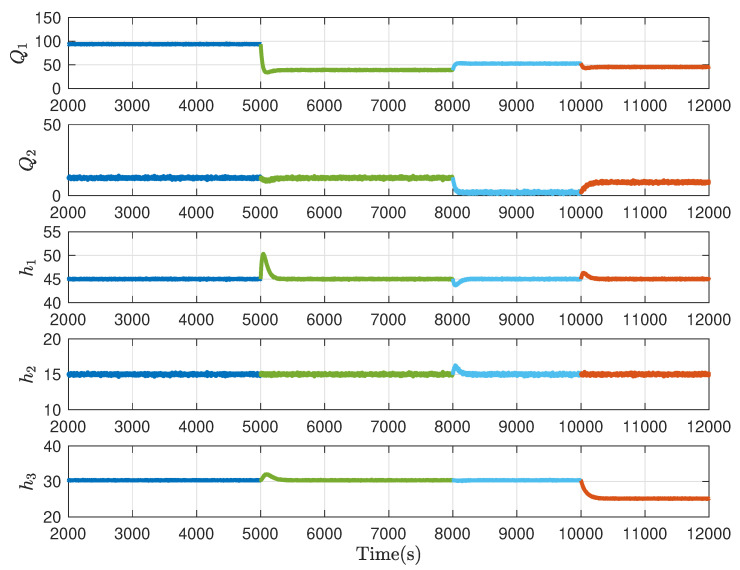
Input and output data.

The parameters of the three-tank model are listed in Table 2. With the linearisation technique, the three-tank system can be formulated in the switched system form (1) with the switching law displayed in the Figure 4. The total running time is 12,000 s and the switching of system modes occurs at 5000 s and 8000 s, respectively. The data of system input and output are collected as shown in Figure 5 with the sampling interval T${}_{s}$ = 2 s.

**Figure 6:**
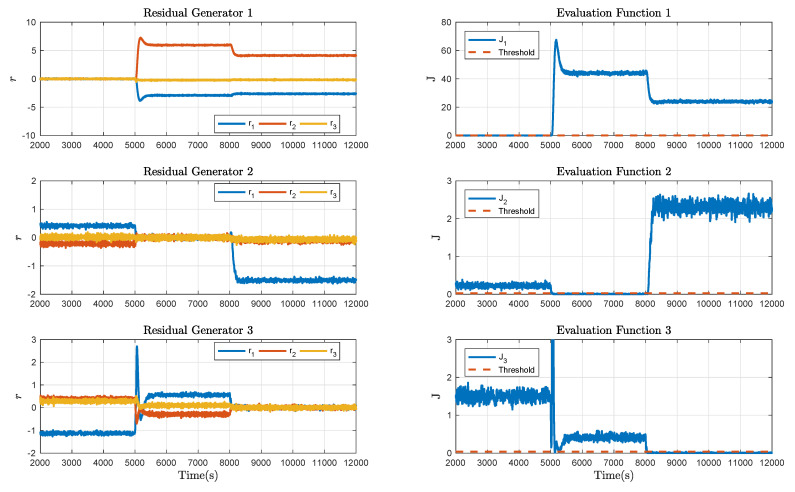
Residual generators, evaluation functions and thresholds designed by Algorithm 1.

**Figure 7:**
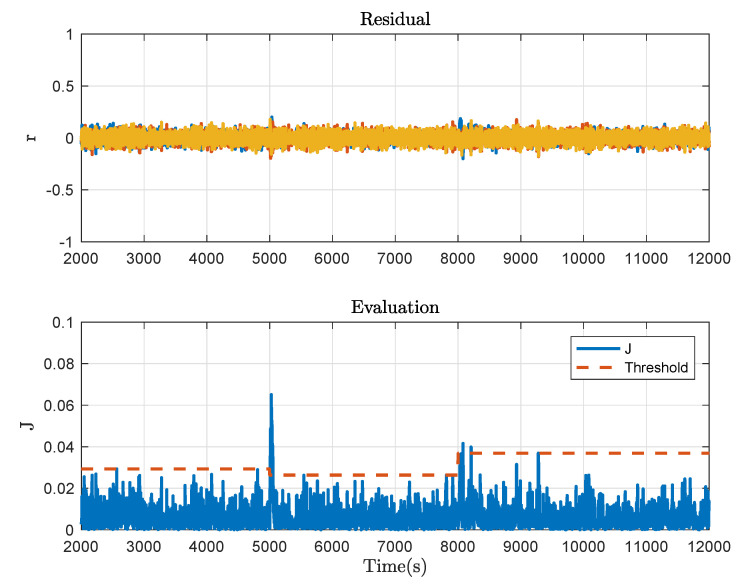
Fault detection result in the fault-free case.

Collect the offline data in the fault-free case and apply Algorithm 1 for each mode. By choosing a proper data length, the Hankel matrices are constructed, based on which the LQ decomposition is implemented and the data-driven SKR is derived. Then the normalised data-driven is calculated via the singular value decomposition. By calculating and comparing the K-gap metric, the residual generators, evaluation functions and thresholds are obtained and exhibited in Figure 6. Observing these curves, it is clear that the three modes are distinguishable. Then the online algorithm, i.e., Algorithm 2, is implemented to show the effectiveness in fault detecting. By collecting the online process data, the residual generator for each mode is presented and further, the residual signals and evaluation function values when there is no fault occurring in the three-tank system are obtained as displayed in Figure 7, from which one can see that the false alarm rate is extremely low. In other words, the designed decision logic improves the mode matching of system plant and residual.

**Figure 8:**
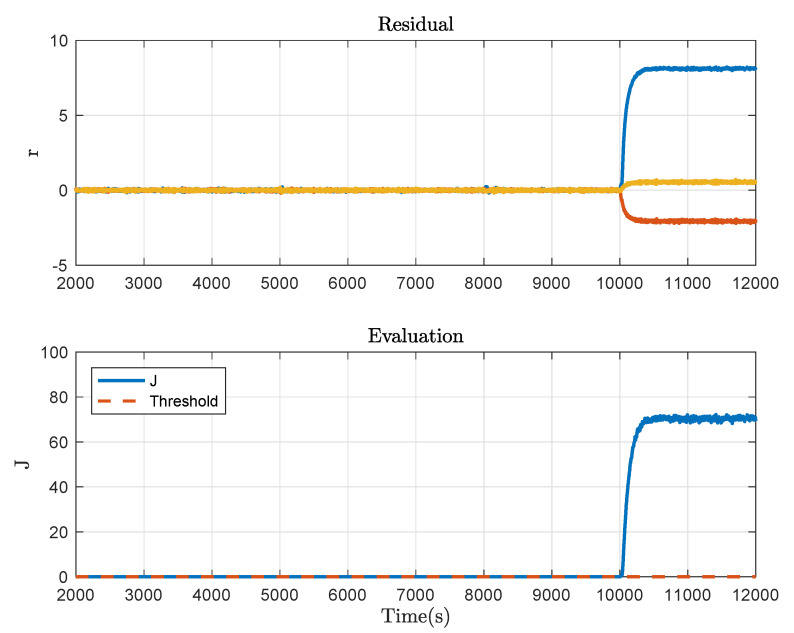
Fault detection result under the plugging fault.

**Figure 9:**
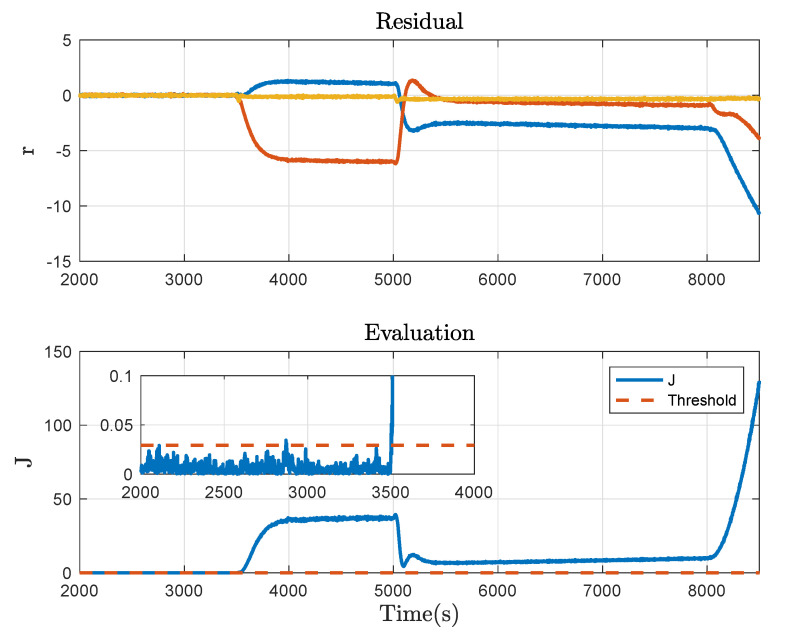
Fault detection result under the offset fault.

**Figure 10:**
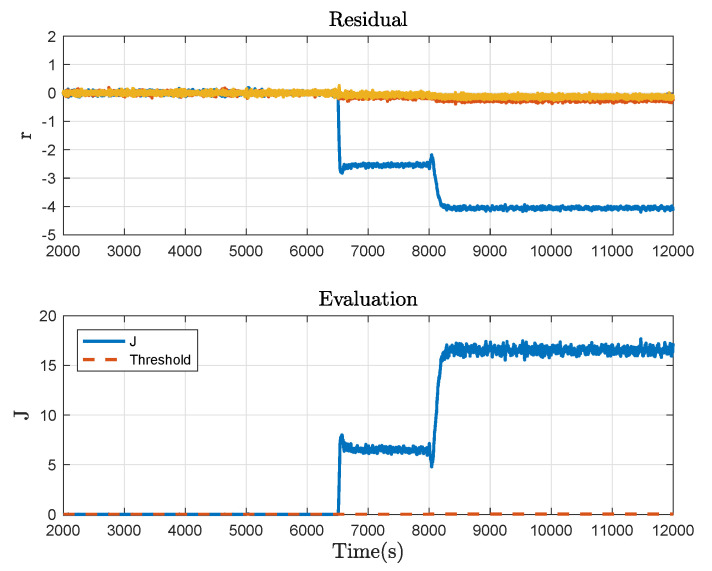
Fault detection result under the leakage fault.

Then consider the system with the plugging fault at 10,000 s, which is caused by closing the valve PV${}_{1}$ by about 30%. Correspondingly, the fault detection result is shown in Figure 8. Moreover, a 5% offset fault in the liquid level sensor of Tank 2 at 3500 s and a leakage fault caused by opening the valve LV${}_{3}$ by about 20% at 6500 s are taken into account. The fault detection results corresponding to the two cases are respectively given in Figure 9 and Figure 10. It can be concluded from these figures that Algorithm 2 effectively detects the fault in progress and thus, the usefulness of the proposed method is demonstrated.

## 5. Conclusions

In this paper, a data-driven approach is developed for the fault detection of discrete-time switched system. Considering the difficulties in acquiring the switching laws in many practical applications, this paper assumes that the switching laws are unavailable to the mode-dependent residual generators in the fault detection implementation. The unavailable switching information may lead to the mode mismatching between system plant and residual generator, for which the mode recognition is considered in this work. Firstly, sufficient criteria are constructed to ensure that the modes of the switched system are distinguishable. Then a decision logic including the mode recognition is presented for the fault detection. Furthermore, offline and online algorithms are elaborated to show the data-driven realisation of the fault detection for the underlying system. A three-tank benchmark system is studied to show the effectiveness of the methods proposed in this paper.

It is worth mentioning that the main contribution of this work is to achieve the data-driven fault detection for switched systems even without available switching laws, which is of great significance from the viewpoint of practice. Actually, the mode recognition approach proposed in this paper is effective in dealing with the analysis and synthesis problems for switched systems with unavailable switching laws and could be further applied to some other research topics of switched systems. In the future, we would like to extend our research to fault-tolerant control for switched systems based on the fault detection method of this work.

## Figures and Tables

**Table 1:** The three modes of the three-tank system.

Mode	PV${}_{1}$	PV${}_{2}$	PV${}_{3}$	LV${}_{1}$	LV${}_{2}$	LV${}_{3}$
Mode 1:	open	open	open	open	close	close
Mode 2:	open	open	open	close	close	close
Mode 3:	open	close	open	open	open	close

**Table 2:** Parameters of the three-tank model.

Parameters	Symbol	Value	Unit
Cross section area of tanks	A	154	cm${}^{2}$
Cross section area of pipes	$s_{n}$	0.5	cm${}^{2}$
Cross section area of drain pipes	$s_{l}$	0.5	cm${}^{2}$
Max. height of tanks	$H_{max}$	62	cm
Max. flow rate of pump 1	$Q_{1_{max}}$	100	cm${}^{3}/s$
Max. flow rate of pump 2	$Q_{2_{max}}$	100	cm${}^{3}/s$
Coeff. of flow for pipe 1	$\alpha_{1}$	0.46	
Coeff. of flow for pipe 2	$\alpha_{2}$	0.60	
Coeff. of flow for pipe 3	$\alpha_{3}$	0.45	
Coeff. of flow for drain pipe 1	$\beta_{1}$	0.46	
Coeff. of flow for drain pipe 2	$\beta_{2}$	0.60	
Coeff. of flow for drain pipe 3	$\beta_{3}$	0.45	

## Data Availability

The data used to support the findings of this study are available from the corresponding author upon request.
